# A New Sparse Adaptive Channel Estimation Method Based on Compressive Sensing for FBMC/OQAM Transmission Network

**DOI:** 10.3390/s16070966

**Published:** 2016-06-24

**Authors:** Han Wang, Wencai Du, Lingwei Xu

**Affiliations:** 1 College of Information Science & Technology, Hainan University, Haikou 570228, China; hanwang1214@126.com; 2 Faculty of International Tourism and Management, City University of Macau, Macau 999078, China; 3 Department of Information Science & Technology, Qingdao University of Science & Technology, Qingdao 266061, China; gaomilaojia2009@163.com

**Keywords:** FBMC/OQAM, channel estimation, compressive sensing, sparse adaptive, greedy algorithm

## Abstract

The conventional channel estimation methods based on a preamble for filter bank multicarrier with offset quadrature amplitude modulation (FBMC/OQAM) systems in mobile-to-mobile sensor networks are inefficient. By utilizing the intrinsicsparsity of wireless channels, channel estimation is researched as a compressive sensing (CS) problem to improve the estimation performance. In this paper, an AdaptiveRegularized Compressive Sampling Matching Pursuit (ARCoSaMP) algorithm is proposed. Unlike anterior greedy algorithms, the new algorithm can achieve the accuracy of reconstruction by choosing the support set adaptively, and exploiting the regularization process, which realizes the second selecting of atoms in the support set although the sparsity of the channel is unknown. Simulation results show that CS-based methods obtain significant channel estimation performance improvement compared to that of conventional preamble-based methods. The proposed ARCoSaMP algorithm outperforms the conventional sparse adaptive matching pursuit (SAMP) algorithm. ARCoSaMP provides even more interesting results than the mostadvanced greedy compressive sampling matching pursuit (CoSaMP) algorithm without a prior sparse knowledge of the channel.

## 1. Introduction

Filter bank multicarrier(FBMC) techniques have drawn increasing attention from many researchers [1,2,3]. In recent years,it has become a competitive alternative to the most famous and accepted Orthogonal Frequency Division Multiplexing (OFDM) schemes, particularly in wireless communication systems. As a potential candidate multicarrier modulation scheme for next generation wireless communication networks [4,5,6,7,8], filter bank multicarrier with offset quadrature amplitude modulation (FBMC/OQAM) is a particular type of FBMC. It utilizes time frequency localization (TFL) well and it employs a property pulse shaping based filter [9] bank, which has a theoretically higher spectral efficiency [10,11]. FBMC/OQAM also demonstrates robustness to frequency offset and Doppler spread. Besides, CP is not needed in FBMC/OQAM systems, which can provide higher data rates than conventional OFDM [12]. FBMC/OQAM system has its root in the pioneering works of Chang [13] and Saltzberg [14] who introduced multicarrier techniques over two decades ago. However, with the conventional OFDM transmission complex-valued symbols in a given symbol rate, FBMC/OQAM transmits real-valued symbols at the symbol rate of two times. The subcarrier functions are only orthogonal in the real field, therefore, an inherent imaginary interference among neighboring subcarriers and symbols is always existed.

The intrinsic inter-carrier/inter-symbol interference will complicate channel estimation processing. Hence, the existing OFDM channel estimation methods cannot be directly applied in FBMC/OQAM systems. Many training schemes and related estimation methods have been recently researched in literature [15,16,17,18]. The two classical preamble-based methods are interference approximation method (IAM) [15,16] and interference cancellation method (ICM) [17,18]. They can be summed up as aiming at avoiding the intrinsic interference or constructively utilizing it to improve the estimation performance. However, it has been proved that the performance of estimator utilizing preamble-based method is inefficient since it is difficult to fully avoid intrinsic interference.

A number of efforts have been devoted to improving the performance of channel estimation. A coded auxiliary pilot channel estimation method for FBMC/OQAM has been proposed [19]. The method is using coded auxiliary pilot symbols to eliminate the imaginary interference on each scatted pilot. Semi-blind and blind symbol timing estimation methods for FBMC/OQAM system have also been studied [20,21]. However, these schemes have a higher computational complexity, and the phase ambiguity may occur and need longer observation data, which, to some extent, limits the availability. A more attractive approach to obtain well channel estimation performance is the recently researched compressive sensing (CS) method [22,23,24,25,26,27], where the wireless channels in practice tend to exhibit a sparse multipath structure. Some channel estimation based on CS methods for OFDM systems have been studied in the past few years [28,29,30,31]. However, there are only few literatures about CS-based channel estimation for FBMC/OQAM systems. An improved IAM that reconstructs channel impulse response by utilizing the orthogonal matching pursuit (OMP) algorithm based on channel estimation for FBMC/OQAM has been proposed in [32]. It is proved that the OMP [33] based method can get remarkable performance improvement compared with the conventional preamble based method. For the most greedy CS algorithms, such as OMP and compressive sampling matching pursuit (CoSaMP) [34], the sparsitylevel of the channel is given as a priori information. However, the sparsity of the channel is usually unknown in most practical application scenarios.

In this paper, a novel channel estimation method named sparse adaptive regularized compressive sampling matching pursuit (ARCoSaMP) is proposed for FBMC/QOAM transmission networks. To the best of our knowledge, sparse adaptive CS-based channel estimation approach has not yet been studied for FBMC/OQAM systems. The advantage of the proposed ARCoSaMP method is that it does not need priori channel sparse information. Furthermore, the proposed algorithm is based on the idea of regularization and the backtracking mechanism that attaches to CoSaMP algorithm, which removes the unreliable support and refines the current approximation iteratively. Simulations verify the proposed channel estimation scheme performs better than the conventional SAMP algorithm and the proposed algorithm can obtain an approximate performance compared with the CoSaMP algorithm.

The purpose of this paper is to propose an efficient sparse adaptive channel estimation method. We would like to convince the reader with the potential of the proposed method as a high performance channel estimator.

The remainder of this paper is organized as follows. The FBMC/OQAM transmission system model is described in Section 2. Section 3 reviews some conventional channel estimation methods (including preamble-based methods and conventional CS recovery algorithms) and presents the proposed scheme. In Section 4, the performances of the proposed scheme associated with the conventional preamble-based and CS-based schemes are compared and simulation results are shown. Finally, Section 5 gives the concluding remarks.

## 2. System Model

In FBMC/OQAM systems, the transmitted signal is given in the following form [15]:

(1) $$x(t)=\sum_{m=0}^{N-1}\sum_{n}d_{m,n}g_{m,n}(t)$$

where $d_{m,n}$ are real valued OQAM symbols, and $g_{m,n}(t)$ denotes the synthesis basis, which can be obtained by the prototype function g(t) in the following way:

(2) $$g_{m,n}(t)=g(t-n\tau_{0})e^{i2\pi mF_{0}t}e^{j\phi_{m,n}}$$

where N is an even number of sub-carriers, the sub-carrier spacing of $F_{0}=1/T_{0}=1/2\tau_{0}$, and $\phi_{m,n}$ an additional phase term. $T_{0}$ denotes OFDM symbol duration, and $\tau_{0}$ denotes the time offset between the real and imaginary parts of the OQAM symbols. m is the sub-carrier index and n is the OQAM symbol time index.

The design of pulse g enables the associated sub-carrier functions $g_{m,n}$ to be orthogonal in the real field,

(3) $$ℜ\{\langle g_{m,n}|g_{p,q}\rangle \}=ℜ\{\sum_{t}g_{m,n}(t)g_{p,q}^{*}(t)\}=\delta_{m,p}\delta_{n,q}$$

where $\delta_{i,j}$ denotes Kronecker delta, $\delta_{m,p}=0$ if m≠p and $\delta_{m,p}=1$ if m=p. We can find that, even in the distortion-free channel and with perfect time and frequency synchronization, some purely imaginary inter-carrier interference at the output still be existed, thus, we set interference weights

(4) $${\langle g\rangle }_{m,n}^{p,q}=-j\langle g_{m,n}|g_{p,q}\rangle$$

where $\langle g_{m,n}|g_{p,q}\rangle$ denotes a purely imaginary term for (m,n)≠(p,q).

**Figure 1:**
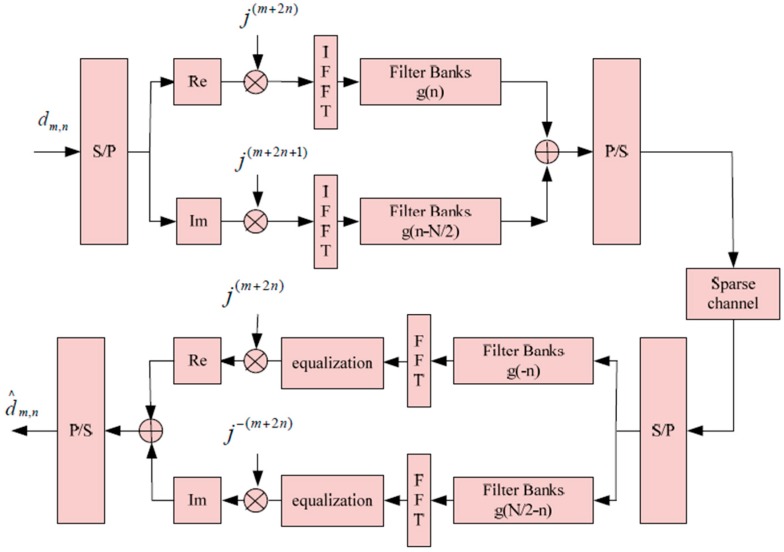
Implementation diagram of the FBMC/OQAM system [35].

Through the channel, with an additive noise, the received signal can be expressed as

(5) $$r(t)=\sum_{m=0}^{M-1}\sum_{n}d_{m,n}g_{m,n}(t)H_{m,n}(t)+\eta (t)$$

with

(6) $$H_{m,n}(t)=\int_{0}^{\tau_{\max}}h(t,\tau )e^{-2j\pi mF_{0}\tau }d\tau$$

where h(t,τ) denotes the channel impulse response, and $H_{m,n}(t)$ denotes a complex response of the channel at instant t. Figure 1 shows an implementation diagram of the FBMC/OQAM system.

## 3. Compressive Sensing Based Preamble Channel Estimation 

In this section, we present a novel sparse adaptive channel estimation method based on CS for FBMC/OQAM systems. We first review the two classical preamble structures and CS theory for channel estimation. We briefly introduce the conventional CS signal recovery algorithms, OMP, CoSaMP and SAMP [36]. Then, we propose the new CS algorithm. Along with the algorithm process, we present numerical evidence showing that our proposed algorithm provides attractive results.

### 3.1. Preamble Structures

**Figure 2:**
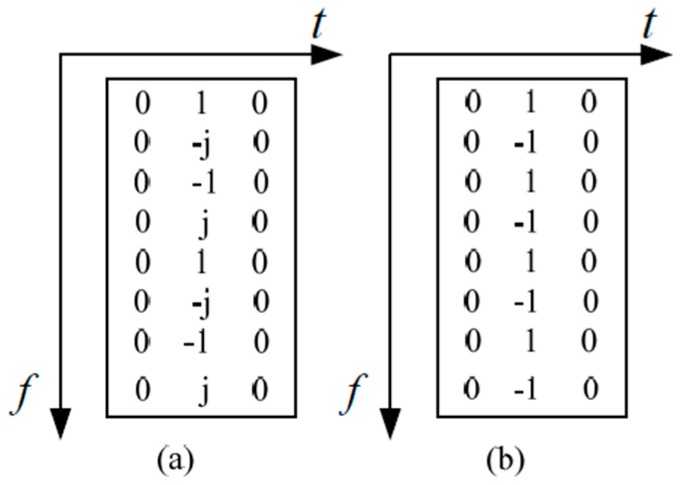
Preamble structures: (**a**) Interference approximation method (IAM); (**b**) Interference cancellation method (ICM).

In FBMC/OQAM, the preamble pilots exist in all sub-carriers, the preamble sequence is superimposed on the data. Figure 2a,b show the IAM and ICM preamble structures. Assuming that a pilot symbol $P_{m_{0},n_{0}}$ is transmitted at a prior known position $\left(m_{0},n_{0}\right)$ to the receiver, the LS estimation is

(7) $${\hat{H}}_{m_{0,}n_{0}}=\frac{r_{m_{0,}n_{0}}}{P_{m_{0},n_{0}}}=H_{m_{0,}n_{0}}+j\sum_{\left(m_{0,}n_{0})\neq (m,n\right)}\frac{d_{m,n}}{P_{m_{0},n_{0}}}{\langle g\rangle }_{m,n}^{m_{0,}n_{0}}$$

where $j\sum_{\left(m_{0,}n_{0})\neq (m,n\right)}\frac{d_{m,n}}{P_{m_{0},n_{0}}}{\langle g\rangle }_{m,n}^{m_{0,}n_{0}}$ is imaginary interference.

### 3.2. CS Theory for Channel Estimation

CS theories [37,38,39] state that a sparse signal h can be recovered steadily from linear measurement

(8) y=Φh+η

where Φ is the measurement matrix, and denotes the matrix with M×N; here, M≪N, and η denotes the noise, by minimizing the $\ell_{1}$-norm of h. The prerequisite is that Φ satisfies the Restricted Isometry Property (RIP), that is, for all K-sparse signal h,

(9) $$1-\delta_{K}\leq \frac{{∥\Phi h∥}_{2}^{2}}{{∥h∥}_{2}^{2}}\leq 1+\delta_{K}$$

where $\delta_{K}$ is the RIP parameter, $0<\delta_{K}<1$.

The signal r(t) in Equation (5) can be given in matrix form as [31]

(10) R=XH+Φ

where X=diag(x(0),x(1),...,x(N−1)), $R={\left[r(0),r(1),...,r(N-1)\right]}^{T}$, and H denotes the multipath channel frequency response sampling value, $H=F_{NL}h$. $F_{NL}$ denotes a L row discrete Fourier Transform matrix, L is the channel length; and Φ denotes the noise matrix, with zero mean and variance of $\sigma^{2}$, and the matrix with N×N.

We set P for the number of pilots, $\varphi =\left(e_{s_{1}},e_{s_{2}},\ldots ,e_{s_{P}}\right)$ denotes a pilot selection matrix with P×N, and φ is utilized to seeking the pilots position from the whole sub-carriers. $s_{i}\left(i=1,2,\ldots ,P\right)$ indicate the ith pilot’s position. Rewrite Equation (10) as

(11) $$R_{P}=X_{P}F_{p}h+Z_{P}$$

where $R_{P}=\varphi R$ is received pilot signal; in this paper, $R_{P}$ is the LS estimation channel values, $\Phi_{P}=\varphi \Phi$, with P column vectors. $F_{P}=\varphi F_{NL}$, and $X_{P}=\varphi X\varphi^{T}$ denotes a diagonal matrix, where the diagonal elements are pilot values.

Let us assume that $F=X_{P}F_{P}$, rewrite Equation (11) as

(12) $$R_{P}=Fh+Z_{P}$$

where h denotes sparse multipath channel response, and we can obtain $R_{P}$ and F in the transmission process. Then, we can use the CS recovery algorithm to recover sparse signal h.

### 3.3. Adaptive Regularized Compressive Sampling Matching Pursuit Algorithm

#### 3.3.1. CS Algorithms Overview

A number of CS recovery algorithms have been proposed. One of the popular kinds of recovery algorithms is based on the iterative greedy pursuit. OMP, CoSaMP and SAMP belong to this class. According to whether the sparse K is known prior or not, this class of algorithms also can be divided into two types.

OMP and CoSaMP are the first type of algorithms with the sparsity *K* is known prior. For the OMP algorithm, in each iteration, the atom maximizes its inner product with the residual signal. However, the results of each iteration may be suboptimal. CoSaMP is proven to be the most advanced greedy algorithm. CoSaMP introduces the idea of backtracking that reduces the chance of error accumulation, selects 2K coordinates and utilizes an iterative checking to refine them, and overcomes the defects of OMP, so that the atoms could not be changed once deposited in the candidate set.

In practical applications, the second type of algorithms has better prospects than the first. SAMP is the second type of algorithm. The sparsity of signal is not required in SAMP as an a priori condition, and SAMP attempts to evaluate the sparsity of source signal by iteration.

#### 3.3.2. Proposed Algorithm

CoSaMP algorithm can reconstruct source signals with high efficiency. However, the algorithm requires the prior knowledge of sparsity. SAMP algorithm provides a way for blind sparse reconstruction. Motivated by the advantages in the two greedy algorithms and associated with a regularized process, we propose a new greedy algorithm, named sparse adaptive regularized compressive sampling matching pursuit (ARCoSaMP).

The proposed algorithm can automatically adjust the selected atoms to reconstruct the unknown sparsity signal in the iterative process. A similar backtracking theory of CoSaMP is utilized to reconstruct partial information of the target signal in the iterative process. An iterative process is divided into multiple stages, the proposed algorithm adaptively estimates the sparsity with steps through stage by stage and set it to the length of the initial support, then gets the accurate target signal by regularization screening of atoms in every stage. The algorithm basic steps are shown below:

**Input**: measurement matrix Φ, the measurement vector y, the initial step size s
**Output:** a K-sparse approximation $\hat{h}$ of the channel h

(1) Initialization: residual r=y, iterative it=1, initial step s=1, stage=1, index value set I=ϕ, J=ϕ.
(2) Set a threshold value ε, if the reconstruction $\hat{h}$ satisfies ${∥h-\hat{h}∥}_{2}\leq \varepsilon$, and stop the iteration; otherwise, continue to Step 3. The deviation norm 2 is chosen as the basis of the iterative termination. In the simulation, $\varepsilon =10^{-7}$.
(3) Calculate the correlation coefficient u by the Equation (13), which calculates the absolute value of inner product between residual r and each atom of measurement matrix Φ, and deposit the index values corresponding the 2s maximum values from u to J:
  (13) $$u=\left\{u_{j}|u_{j}=\left|\langle r,\Phi_{j}\rangle \right|,j=1,2,\cdot \cdot \cdot ,N\right\}$$
(4) Regularization: using Equation (14) to regularize
  (14) $$\left|u(i)\right|\leq 2\left|u(j)\right|\text{for all }i,j\in J_{0}$$
  choose $J_{0}$ with the maximal energy ${∥u{|}_{J_{0}}∥}_{2}$, add the set $J_{0}$ to the index set I, and update the support set $\Phi_{I}$.
(5) Use Equation (15) to get $\hat{h}$, according to the backtracking mechanism, take atoms corresponded to the largest s elements of $\hat{h}$ to I, update the support set $\Phi_{I}$:
  (15) $$\hat{h}=\arg\min{∥y-\Phi_{I}h∥}_{2}$$
(6) Update the residual
  (16) $$r_{new}=y-\Phi_{I}\hat{h}$$
(7) Make a comparison between the update residual and the last iteration residual; if ${∥r_{new}∥}_{2}\geq {∥r_{n-1}∥}_{2}$, stage=stage+1, s=s⋅stage, return to Step 3; otherwise, $r=r_{new},\;n=n+1,$ and return to Step 2.

The selection of the initial step is very important, and if the step size is too large, there may an overestimating problem. In the proposed algorithm, the initial step size is 1, which is less than the reality of the sparsity K until the final stage. Iteration loop follows the CoSaMP and regularization to identify support sets in the target signal. When s<K, it is necessary to take an effective mechanism for stage switching. In the proposed algorithm, we trigger the stage switching between two consecutive iterations when the relevant residual improvement begins to disappear.

## 4. Simulation Results

In this section, simulation results are presented to compare the performance between convention LS, OMP, CoSaMP, SAMP and the proposed algorithm. The evaluations are mainly based on bit error rate (BER) and mean square error (MSE). The MSE is plotted with respect to the signal to noise ratio (SNR). The estimation of multipath delay profile and percentage recovered of the algorithms are also given. We take modulation as 4OQAM, the number of subcarriers in FBMC/OQAM systems is N=2048. The square root raise cosine filter is employed in FBMC/OQAM, the roll off factor of the filter is one, and length of the filter is $4T_{0}$ We adopt the IEEE 802.22 channel with sampling frequency 6.86 MHz as a simulation channel. The channel profile is shown in Table 1. The channel sparse *K* is 6. The channel coding adopts convolutional code (k=7 with $g_{1}={\left(133\right)}_{o},\;g_{2}={\left(171\right)}_{o}$ and code rate = 1/2).

**Figure 3:**
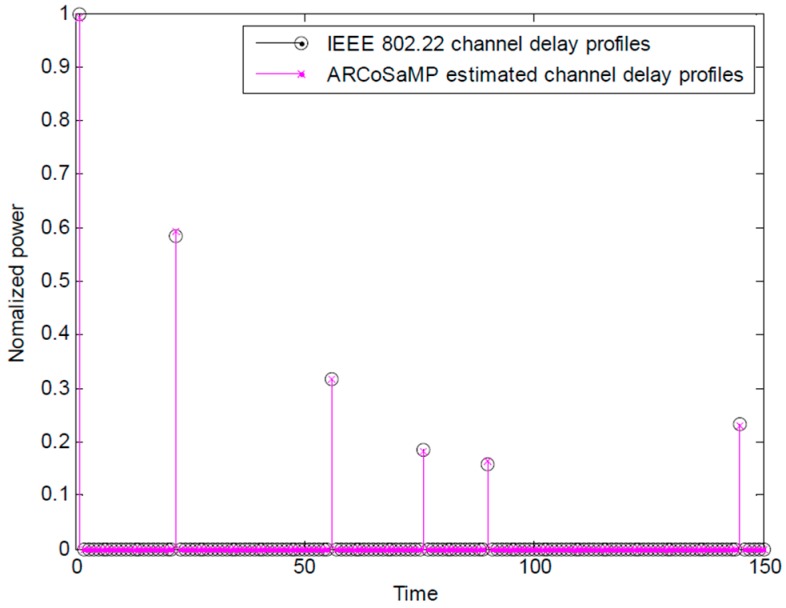
IEEE 802.22 channel delay profile and estimated channel delay profile.

Figure 3 is a snapshot of the original and estimated delay profiles of IEEE 802.22 channel. It shows that the proposed ARCoSaMP for FBMC/OQAM successfully detect the channel with sixmultipaths and SNR = 8 dB. The proposed scheme not only precisely estimates the multipath delay values but also exactly estimates the relative power of the multipath.

**Figure 4:**
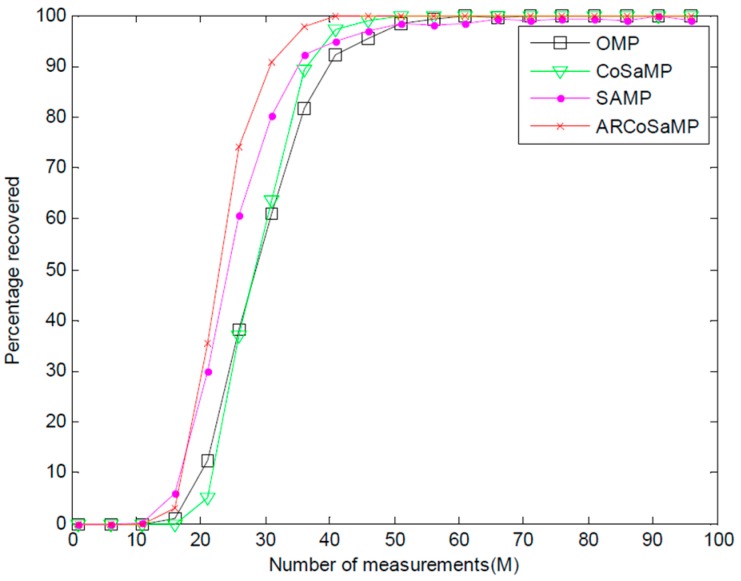
Probability of exact recovery versus the number of measurements.

We also investigate the probability of recovery for a fixed signal sparsity K=6 among the above-mentioned algorithms. Figure 4 depicts the probability curves of Gaussian sparse signal. It can be seen that ARCoSaMP outperforms the other three algorithms in the range of M=15 to M=35. ARCoSaMP also requires the least measurements for exact recovery. When the measurements M>35, CoSaMP provides better recovery probability than SAMP. With the measurement increasing, ARCoSaMP provides recovery probability approximate to that in CoSaMP without prior knowledge of sparsity.

**Figure 5:**
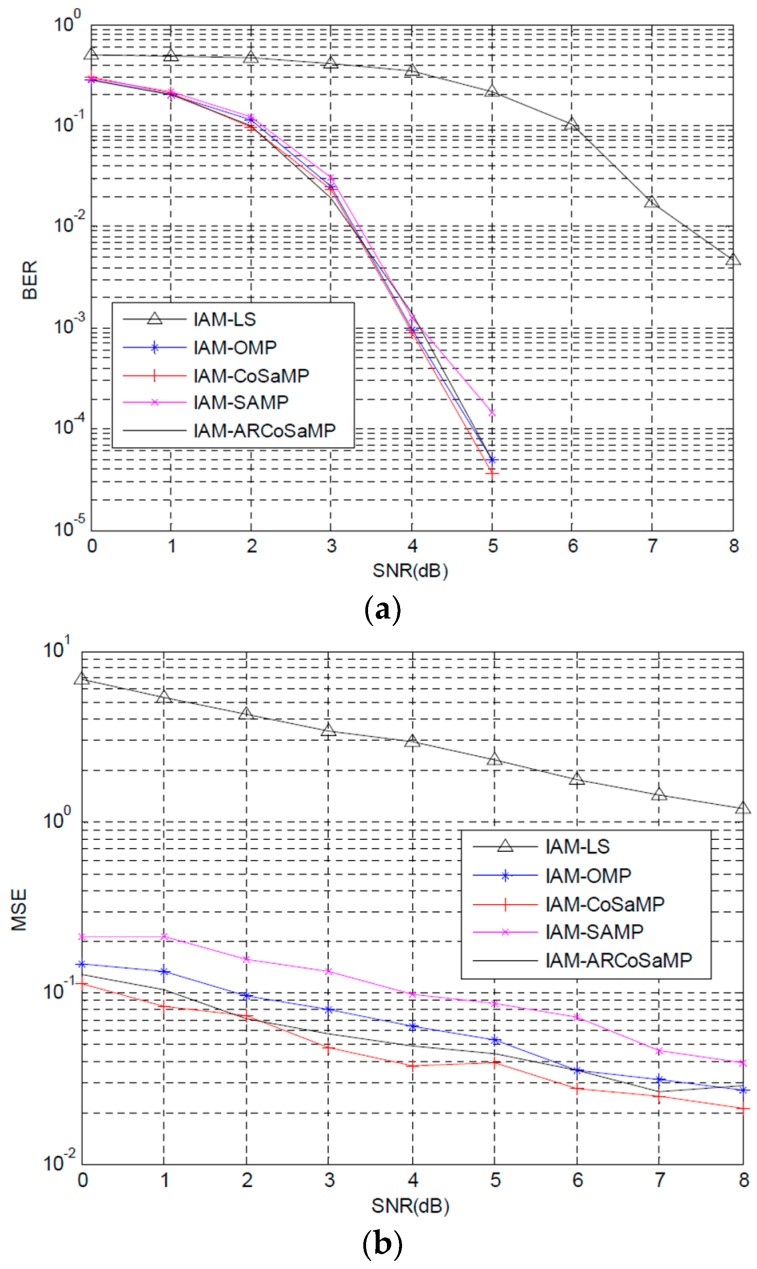
Bit error rate (BER) and mean square error (MSE) performance comparisons based IAM preamble structure: (**a**) BER and (**b**) MSE.

Figure 5a,b shows the BER and MSE performance comparisons when IAM preamble structure is adopted in FBMC/QOAM systems. In Figure 5a, it is obvious that CS based channel estimation methods can obtain significantly BER improvement compared with conventional least squares (LS) method. Careful observation shows that CoSaMP outperforms other algorithms in the whole SNR range considered. CoSaMP can obtain about 4.2 dB gain compared with LS when BER of $10^{-2}$ is considered. ARCoSaMP performs slightly better than OMP, and has a gain of 0.3 dB compared with SAMP, when the BER of about $10^{-4}$ is considered. Figure 5b plots the MSE performance comparisons. We can see that CoSaMP still performs the best MSE performance in the five schemes. It enjoys a significant SNR gain compared to those of LS, when at the same MSE level. ARCoSaMP is slightly worse performing than CoSaMP, but well performing than OMP and SAMP. It should be noted that ARCoSaMP provides BER performance comparable to CoSaMP algorithm without the prior known of sparsity and exceeds the conventional SAMP algorithm. In addition, we compare simulation time of SAMP and ARCoSaMP, where both of the two algorithms do not need to know the prior knowledge of sparisity. The result shows that ARCoSaMP needs less simulation time than SAMP, with ARCoSaMP simulation time is 30.1824 s and SAMP simulation time is 32.8995 s.

**Figure 6:**
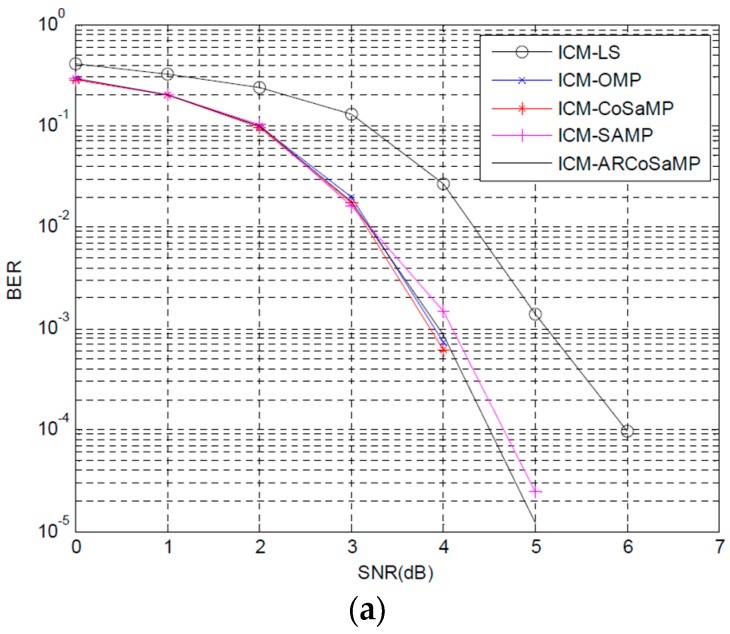

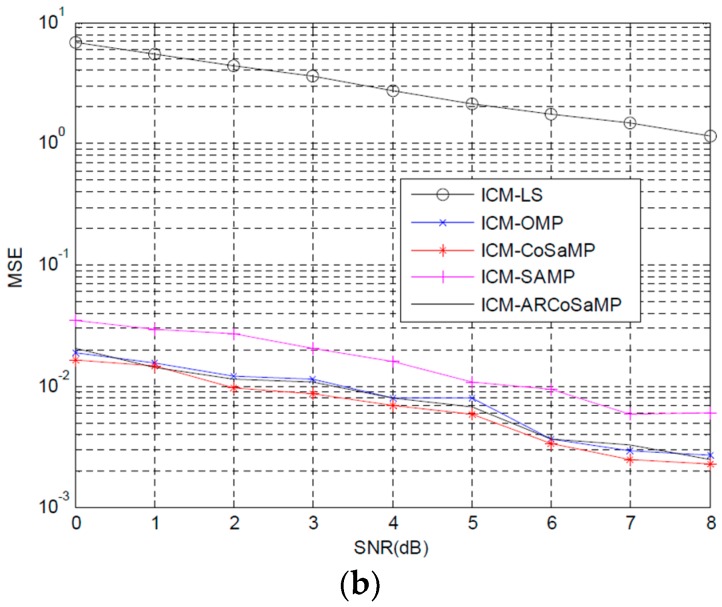
BER and MSE performance comparison based ICM preamble structure: (**a**) BER and (**b**) MSE.

Figure 6a,b depict the BER and MSE performance comparisons when ICM preamble structure is adopted in FBMC/QOAM systems. We can find that the trends of both BER and MSE curves are the same as that in Figure 5. Preamble structure ICM based channel estimation outperforms the IAM scheme. ICM-LS provides significant BER improvement compared with IAM-LS. ICM-CS has slight BER improvement compared with IAM-CS but obvious improvement in MSE performance. CoSaMP algorithm obtains the best BER and MSE performances. In Figure 6a, ARCoSaMP gives performance that are about 0.2 dB better than SAMP method, when BER = $10^{-2}$. CoSaMP can obtain about 1.2 dB gain compared with LS when BER of $10^{-2}$ is considered. In Figure 6b, the OMP, CoSaMP and ARCoSaMP, the three curves, are very close. ARCoSaMP and OMP provide similar MSE performance, and CoSaMP is less well performing than ARCoSaMP. The simulation times of ICM-SAMP and ICM-ARCoSaMP are less than IAM-SAMP and IAM-ARCoSaMP. ICM-ARCoSaMPstill needs less time than ICM-SAMP.

As shown in the simulation results, it can be verified that the CS-based channel estimation approach can provide more effective performance than conventional LS methods in FBMC/OQAM systems. The proposed ARCoSaMP based channel estimation method can achieve similar performance than CoSaMP without a prior sparse knowledge of the channel and has better channel estimation performance than SAMP with lesser time complexity.

## 5. Conclusions

In this paper, we have studied the preamble channel estimation based on compressive sensing for FMBC/OQAM systems under an IEEE 802.22 sparse multipath channel. A new sparse adaptive regularized compressive sampling matching pursuit algorithm for channel estimation is proposed, which is associated with adaptive, regularized and CoSaMP. The proposed algorithm can accurately estimate the multipath components. Simulation results demonstrate that a CS based preamble approach can achieve significantly better BER and MSE performance than conventional LS methods. The proposed scheme outperforms SAMP for channel estimation with lesser time complexity and can provide approximate results than the state-of–the-art CoSaMP algorithm without a prior sparse knowledge of the channel. It has been verified that the ARCoSaMP scheme is an efficient method for sparse adaptive channel estimation in FBMC/OQAM transmission networks.

## Figures and Tables

**Table:** 

Multi-Paths	1	2	3	4	5	6
Delay(μs)	0	3	8	11	13	21
Power(dB)	0	−7	−15	−22	−24	−19

**Table 1:** IEEE 802.22 channel profile.

Multi-Paths	1	2	3	4	5	6
Delay(μs)	0	3	8	11	13	21
Power(dB)	0	−7	−15	−22	−24	−19
